# High Hydrostatic Pressure vs. Thermal Pasteurization: The Effect on the Bioactive Compound Profile of a Citrus Maqui Beverage

**DOI:** 10.3390/foods10102416

**Published:** 2021-10-12

**Authors:** Francisco J. Salar, Paula M. Periago, Vicente Agulló, Cristina García-Viguera, Pablo S. Fernández

**Affiliations:** 1 Phytochemistry and Healthy Foods Lab (LabFAS), Department of Food Science and Technology, (CEBAS-CSIC), University Campus of Espinardo, Edif. 25, 30100 Murcia, Spain; fjsalar@cebas.csic.es (F.J.S.); vagullo@cebas.csic.es (V.A.); 2 Agronomic Engineering Department, Universidad Politécnica de Cartagena (UPCT), Paseo Alfonso XIII, 48, 30203 Cartagena, Spain; paula.periago@upct.es (P.M.P.); pablo.fernandez@upct.es (P.S.F.); 3 Associated Unit of Food Quality and Risk Assessment CEBAS-CSIC/UPCT, 30100 Murcia, Spain

**Keywords:** high hydrostatic pressure, thermal pasteurization, anthocyanin, vitamin C, flavanones

## Abstract

The effects of high hydrostatic pressure (HHP) compared to thermal pasteurization (TP) were studied in healthy citrus-maqui beverages. The impact of the processing technologies on the microbiological and phytochemical profile was assessed by applying two HHP treatments at 450 and 600 MPa for 180 s and TP at 85 °C for 15 s. The shelf life under refrigeration (4 °C) and room temperature (20 °C) was monitored for 90 days. All treatments ensured microbiological stability at both storage temperatures. Aside from that, the physicochemical parameters were not significantly different after processing or throughout the storage period. Regarding color parameters, an increase in the reddish coloration was observed during storage for those beverages treated by HHP. In general, phenolic compounds were little affected by the processing technique, even when treatment under HHP was more stable than by TP during storage. On the other hand, vitamin C showed great degradation after processing under any condition. It can be concluded that HHP is an effective alternative to thermal treatments, achieving effective microbial inactivation and extending the shelf life of the juices by contributing to a better preservation of color and bioactive compounds.

## 1. Introduction

Nowadays, there is a growing trend focused on the implementation of healthy and balanced diets by consumers, minimizing the consumption of processed foods. In this respect, recent studies revealed that over 70% of consumers associate emerging non-thermal technologies with better nutrient or sensory quality [1], since these novel techniques are capable of maintaining a very similar freshness for unprocessed foods, minimizing losses in the physicochemical and organoleptic profiles and ensuring microbial safety during subsequent storage, as well as being environmentally friendly [2,3]. Moreover, consumers are increasingly aware of including fruit- and vegetable-based products in their diets due to their nutritional importance and potential health-promoting profiles, which can be useful to support the immune system, prevent chronic diseases and micronutrient deficiencies, or counteract or inhibit the progression of degenerative diseases caused by pro-oxidant agents [4]. Consequently, there is a marked increase in the demand for high-quality foods, with attractive organoleptic and nutritional characteristics similar to fresh equivalents, ensuring maximum food safety in addition to being minimally processed and without using preservatives, additives, or colorings [5,6].

This study follows previous studies focused on the design of healthy plant-based soft drinks, which showed that thermally treated beverages based on maqui and citrus fruits are an excellent source of bioavailable bioactive compounds [7,8,9], mainly vitamins and phenolic compounds with wide biological activity [10,11]. This is based on the health-protective properties of berries, namely maqui berry (*Aristotelia chilensis* (Mol.) Stunz), a native plant from Chile and Argentina particularly studied as a potential healthy food ingredient [12,13]. The functional capacity of this berry has been related to its high anthocyanin concentration, which is partially responsible for its antioxidant and anti-inflammatory capacity [14], in addition to cardio or neuroprotective effects [15] as well as preventing chronic degenerative diseases such as obesity [16] or cancer [17,18]. That aside, its contribution to postprandial hyperglycemia decreasing has been pointed out, with inhibitory effects regarding enzymatic activity involved in metabolic syndromes [19] and those effects related to aging and antioxidant activity [20].

On the other hand, citrus fruits are a source of nutraceutical and bioactive phytochemicals [21] such as flavonoids, particularly flavanones, and phenolic acids, in addition to vitamins, carotenoids, minerals, dietary fiber, and essential fatty acids that provide antimicrobial [22], anti-inflamatory [23,24], and antitumor [25] functions, as well as biological activity against obesity [26], diabetes [27], and neuroprotective [28] and cardioprotective effects [29].

Concerning the different beverage processing methods, conventional thermal methods have been capable of ensuring food safety and achieving a high degree of enzymatic inactivation in the fruit- and vegetable-based beverage industry. However, thermal processing (TP) tends to degrade the overall quality in juices, such as the sensory and phytochemical profiles [30], affecting the functional capacity of the drinks. This general loss of quality is directly related to consumer acceptance, resulting in recent years in a substantial stagnation and decrease of processed beverages with thermal treatments in comparison with minimally or non-thermally processed beverages, which are rapidly expanding worldwide [31,32]. Currently, the primary new non-thermal technologies that are on the rise are high hydrostatic pressure (HHP), pulsed electric fields (PEF), ultrasound (US), ultraviolet light (UV), and cold plasma, among others [33]. In this sense, HHP has achieved the greatest success [34], with a continuously expanding market due to its capacity to reduce the microbial load of both pathogens and disruptive microorganisms in multiple juice matrices, consequently resulting in an extension of the storage period [35] while preserving the organoleptic, nutritional, and phytochemical characteristics of the juices [36,37]. Due to the intrinsic characteristic of this technology, at moderate temperatures, high pressures do not affect the covalent bonds during the pressurization process [38,39], with food biomolecules minimally affected in vegetable- and fruit-based drinks [40,41]. What is more, an increase in the levels of nutrients and bioactive compounds has been reported in HHP-treated juices, as this technology would enhance the extractability of these substances [42]. In addition, HHP at pressures usually >400 MPa would also assist to inhibit, totally or in most part, the endogenous enzyme activities of cases only partially, which are responsible for undesired changes in the global quality of the juices in storage [43]. On this basis, fruit or vegetable HHP beverages generally give rise to a better overall quality than samples treated with conventional heat treatments [44,45].

Given these antecedents, the purpose of the present study was the optimization of the processing design for healthy maqui-citrus soft drinks using HHP in order to minimize the loss of bioactive compounds and, consequently, increase the healthy properties previously reported. Thereby, a comparison between HHP (at 450 and 600 MPa) and TP (under standard industrial processing conditions) for their quality parameters (physicochemical and microbial profiles) and bioactive compound contents (flavonoids and vitamin C) over 90 days of storage at 4 °C and 20 °C was set. Moreover, to our knowledge, this is the first study comparing the effect of high hydrostatic pressure relative to TP on the levels of functional compounds and shelf life in juices containing a mixture of bioactive compounds from berry and citrus fruits.

## 2. Materials and Methods

### 2.1. Chemicals and Reagents

Hesperidin was obtained from Merck (Darmstadt, Germany), and cyanidin 3-*O*-glucoside was purchased from TransMIT (Geiben, Germany). Acetonitrile, formic acid, methanol, and ethylenediaminetetraacetic acid disodium salt 2-hydrate (EDTA), were obtained from Panreac (Barcelona, Spain). Buffered peptone water, Plate Count Agar (PCA), Rose Bengal Agar (RBA), Brilliant Green Bile 2% Broth (BGBB) and Man Rogosa Sharpe Agar (MRSA) were purchased from Scharlab (Barcelona, Spain). L-ascorbic acid (AA) and dehydroascorbic acid (DHAA) were acquired from Acros Oganics (Thermo Fisher Scientific Inc., Madrid, Spain) and Sigma-Aldrich (St. Louis, MO, USA), respectively. All solutions were prepared with ultrapure water from a Milli-Q Advantage A10 ultrapure water purification system (Millipore, Burlington, MA, USA).

### 2.2. Ingredients

Fresh dry maqui powder organic was provided by Maqui New Life S.A. (Santiago, Chile), citrus juices by Cítricos de Murcia S.L. (Ceutí, Murcia, Spain) and AMC Grupo Alimentación S.A. (Espinardo, Murcia, Spain) and sucrose by AB Azucarera Iberia S.L. (Madrid, Spain).

### 2.3. Beverage Preparation

Juice preparation was conducted according to previous studies of the group [7]. Briefly, maqui powder was mixed with citrus juices and sugar to obtain the base beverage.

### 2.4. HHP Processing and Thermal Pasteurization

The juices were poured into PET clear bottles (80 mm, 27.5 mm Ø; vol. 30 mL) with plastic screw caps (Sunbox, Barcelona, Spain) prior to HHP processing and subsequently in the case of TP. The beverages were pressurized using a commercial Hiperbaric 135 for HHP (Hiperbaric, Burgos, Spain). This equipment has a capacity of 135 L, which applies a maximum treatment of 6000 bares (600 MPa). The samples underwent two HHP treatments at 450 and 600 MPa for 3 min at 20 °C. The times to reach 450 MPa or 600 MPa were 120 and 165 s, respectively. The initial water temperature in the vessel ranged from 8–10 °C, and the rate of temperature rise during compression was 3 °C/100 MPa, while the decompression of the beverages was almost instantaneous (less than 5 s). Selection of processing parameters was mainly based on HHP common industrial processing conditions for ensuring microbial safety in juices with low pH levels.

The thermal treatment was carried out with a Mastia thermoresistometer [46]. The initial temperature was 20 °C. The thermoresistometer was programmed with a ramp at a heating rate of 40 °C/min to reach the target temperature of 85 °C. The temperature was held at 85 ºC for 15 s, according to common industrial standards for similar beverage compositions and pH levels. Then, the product was cooled down at a speed of 40 °C/min, reaching a temperature of 20 °C, and stored in the same PET clear bottles as mentioned before. It is important to note that equivalent HHP processing and thermal conditions were selected in terms of industrial microbial safety for an equal comparison of both preservation technologies.

### 2.5. Sampling

All treated beverages were stored immediately after processing at 4 and 20 °C in darkness for 90 days. The samples were labeled according to the codification specified in Table 1, where all the test conditions are included. All juices and experimental conditions tested were prepared in triplicate (*n* = 3), and all analytical determinations were performed in triplicate (*n* = 3). The samples were analyzed at 0, 7, 15, 30, 45, 60, and 90 days. Moreover, untreated samples were stored and analyzed as controls against the treated ones for day 0.

### 2.6. pH, Titratable Acidity, and Total Soluble Solids

The pH values were measured using a GLP 21 pH meter (Crison Ltd., Barcelona, Spain). The TA was determined using a 794 Basic Titrino (Metrohm) by titrating 2 g of juice (up to 30 g of Milli-Q water) with 0.1 mol/L NaOH to an end point of pH 8.1. The results were expressed as grams of citric acid per 100 mL of the sample (g CA/100 mL). The TSS contents of the samples were recorded in a refractometer (Abbe WYA-S, Optic Ivymen^®^ System; Biotech SL, Barcelona, Spain) at 20 °C, with values being expressed as °Brix [7].

### 2.7. Microbiology Analysis

The samples were aseptically diluted in buffered peptone water and then analyzed for aerobic mesophilic bacteria, aerobic psychrophilic bacteria, molds and yeasts, Enterobacteriaceae, and Lactic Acid Bacteria (LAB). They were analyzed at day 0 and after 7, 15, 30, 45, 60, and 90 days of storage at 4 °C and 20 °C. Microbial analyses were carried out in order to measure the short- and long-term effectiveness of HHP processing and TP at day 0 just after processing and throughout 90 days of shelf life. The quantifications of the molds and yeasts were determined by plating the samples in Rose Bengal Agar (RBA), followed by incubation for 5 days at 25 °C. The counts for aerobic mesophilic and psychrophilic bacteria were performed using the Plate Count Agar (PCA) medium, incubated for 48 h at 30 °C and for 10 days at 5 °C, respectively. Enterobacteriaceae were determined using the more probable number method with Brilliant Green Bile 2% Broth (BGBB) after incubation for 24–48 h at 37 °C. Lactic Acid Bacteria (LAB) were determined using Man Rogosa Sharpe Agar (MRSA) after incubation in anaerobic conditions for 5 days at 37 °C. Microbial counts were expressed as colony-forming units per milliliter (CFU/mL).

### 2.8. Qualitative and Quantitative Analysis of Phenolic Compounds by RP-HPLC-DAD

Juice samples were processed following the method previously described [7]. The identification and quantification of phenolic compounds was carried out by applying the method previously reported [7,8]. The diverse phenolic compounds in the samples were identified by comparison with authentic standard compounds of analytical grade. Flavanones were quantified as hesperidin at 280 nm and anthocyanins as cyanidin 3-*O*-glucoside at 520 nm. The concentration of phenolic compounds was expressed as mg per 100 mL of juice.

### 2.9. Extraction and Analysis of Vitamin C

The content of vitamin C was found by applying the UHPLC-ESI-QqQ-MS/MS-based method recently developed [47] and calculated by comparison with freshly prepared ascorbic acid (AA) and dehydroascorbic acid (DHAA) authentic standard curves. The results were expressed as mg per 100 mL of juice.

### 2.10. Color Measurements

The color was determined using a Konica Minolta CM-5 Chroma Meter (Osaka, Japan). The results were expressed in accordance with the CIEL*a*b* system with reference to a visual angle of 10° and a light source set on D65. Three measurements of each sample were performed, and the values were averaged. The color parameters determined were the luminosity (CIEL*), redness (a*), and yellowness (b*) using the CM-5 spectrophotometer in reflection mode. The *Hue* angle (H), *Chroma* (C) and total color differences (ΔE) were calculated from tan^−^^1^ (b*/a*), (a*^2^ + b*^2^)^1/2^, and (da*^2^ + db*^2^ + dL*^2^)^1/2^, respectively [7].

### 2.11. Statistical Analyses

The results are presented as the means ± SD (*n* = 3). A paired *t*-test was developed to compare two parameters, and analysis of variance (ANOVA) and Tukey’s multiple range tests were carried out to compare three or more conditions. All statistical analyses were performed using SPSS 19.0 software (LEAD Technologies, Inc., Chicago, IL, USA). The level of statistical significance was established at *p* < 0.05.

## 3. Results and Discussion

### 3.1. Initial Impact of Proccesing on the Overall Quality Parameters in Juices

In general, neither HHP nor TP presented differences in their pH, acidity, or °Brix values, which remained stable immediately after processing (*p* > 0.05) in all treated drinks (Table 2). Hence, the preservation treatment was not a relevant parameter concerning physicochemical characteristics. These results agree with those previously obtained by Chung et al. and Nayak et al. in fruit juices [48,49].

On the other hand, the samples treated under both conditions exhibited significant color differences (ΔE) in comparison with the untreated beverage (*p* < 0.05) in the aftermath of processing (Table 2). These changes were more prone to thermal treatment, mainly due to a decrease in the L value (lightness) when high temperatures were applied, in concordance with some authors [50], while HHP processing caused minor variations in the drinks’ colors, as previously reported by others [51], although according to Orellana-Palma et al., these differences were not detectable with the naked eye for values of ΔE lower than four units [52].

Regarding flavonoids (anthocyanins and flavanones), slight significant changes (*p* < 0.05) were noticed after all treatments (Table 2). These results indicated that the initial total content of anthocyanins slightly decreased after both HHP conditions for processing, while this content increased up to 11% after TP. These results differ from other authors [53,54], who have reported higher recovery of anthocyanins for processing treatments under pressure in comparison with heat treatments. Moreover, concerning the total content of flavanones, there were significant differences (*p* < 0.05) in the pasteurized juices just after processing related to HHP and the unprocessed beverages. In this sense, TP managed to enhance the content and, consequently, bioaccessibility of these bioactive molecules up to 55% more, while both HHP treatments were capable of maintaining the initial flavanone content at a rather stable value compared with the untreated juice. This increase in flavonoids after thermal treatment may have been associated with the increased extractability of them as they were released when applying moderate temperatures during thermal treatment due to vegetable cell wall disruption, as previously reported [55,56]. It should be noted that the results of this study were in concordance with those found by He et al., who after thermal processing increased the total phenolic content up to 39% in orange juice, mainly due to the individual contribution of hesperetin-rutinoside, which increased its initial value by 27% post-pasteurization [57].

Meanwhile, changes regarding other bioactive compounds such as vitamin C were noticeable between the untreated and treated samples (Table 2). Either pressurized or pasteurized beverages showed lower total vitamin C contents (*p* < 0.05) compared with the unprocessed citrus-maqui drink by 35% on average after all the processes (Table 2). These findings agree with Spira et al., who also reported similar results for HHP and pasteurization treatment in orange juice [58]. In the same context, Andrés et al. also indicated a significant decrease in ascorbic acid immediately after processing for all preservation treatments, with the specimen under HHP treatment (7% loss on average) being lower than that under TP treatment (12% loss on average) in a fruit-based beverage [59].

On the other hand, concerning the microbiological aspects, the untreated samples were characterized by an initial concentration of aerobic mesophilic bacteria of 80 ± 5 CFU/mL (Table 3), lower than the detection limits for molds and yeasts (<100 CFU/mL), and for aerobic psychrophilic bacteria, Enterobacteriaceae and lactic acid bacteria concentrations were less than 10 CFU/mL. Nevertheless, either HHP or TP processing managed to reduce these values below the detection limits immediately after applying treatments for aerobic mesophilic bacteria. Similar results have been previously reported by other authors, who achieved, just after processing by HHP or TP, microbial counts below the detection limits in plant-based beverages [60,61,62].

### 3.2. Effect of HHP and TP Treatments on pH, Tritrable Acidity (TA), and Total Soluble Solids (°Brix) during Storage

According to the data related to the physicochemical parameters obtained in the present work, the total soluble solids (°Brix) values were similar in all treated beverages (*p* > 0.05) (Table 4), with only a slight increase ranging from 13.60 to 14.20 at the end of the storage time. Concerning the pH and total titratable acidity, there were no significant differences between both HHP treatments and TP, remaining rather stable during the monitored storage period. These results are consistent with other authors that have also reported no differences regarding these physicochemical parameters over the storage period in similar juice matrices after processing by HHP or thermal treatment [63,64].

### 3.3. Changes in the Microbiological Profiles during Storage

The microbiological profiles of the citrus-maqui juice samples treated by HHP (450 and 600 MPa/3 min) and TP (85 °C/15 s) were analyzed during storage for 90 days at both 4 and 20 °C. In the current study for all the processed juices, no signs of microbial growth were observed throughout the 90 days of storage, with microbial counts remaining below the detection limit for all microorganisms analyzed, indicating that the citrus-maqui blends were microbiologically safe and stable at both refrigerated and room temperatures. Previous studies of similar technologies have also reported efficacy in preserving microbiological safety over the storage period in vegetable- and fruit-based juices. In this respect, Chen et al. reported that both HHP and thermal treatment ensured microbiological stability during 90 days of storage at 4 °C in pomegranate juice [65]. Moreover, Hsu et al. also pointed out that HHP and TP managed to achieve a microbiologically stable product during 28 days of refrigerated storage in tomato juice [66]. Finally, Bull et al. and Parish indicated that orange juice subjected to HHP or TP was able to maintain its microbiological quality for a shelf life of up to 4 months in chilled storage [67,68].

### 3.4. Effect of HHP and TP Treatments on Vitamin C during Storage

**Figure 1:**
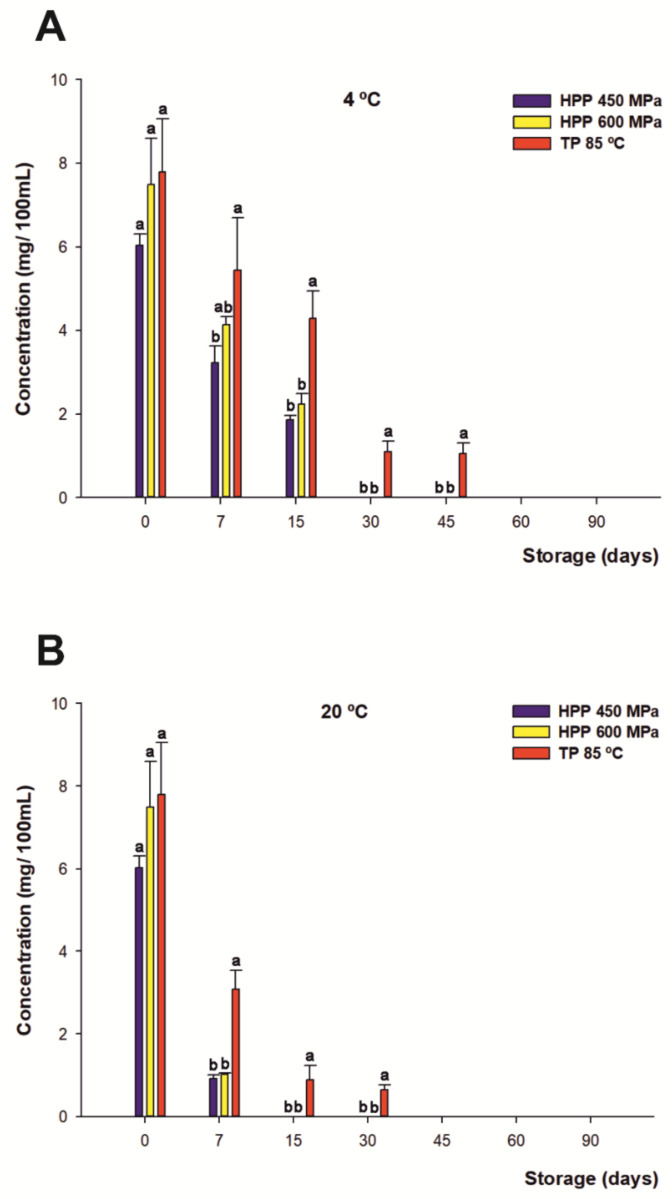
Changes in content of vitamin C (mg/100 mL) for juices subjected to high hydrostatic pressure (HHP—450 MPa and HHP—600 MPa) and thermal pasteurization (TP—85 °C), measured during storage for 90 days under refrigerated conditions at 4 °C (**A**) and 20 °C (**B**) under darkness conditions. Bars with different lowercase letters within each time point were different statistically at *p* < 0.05 according to the analysis of variance (ANOVA) and Tukey’s multiple range test.

The content of vitamin C was mainly due to citrus plants, which are natural sources of this antioxidant, as maqui powder is not a relevant source. It was quantified by UHPLC-ESI-QqQ-MS/MS and reported by Salar et al. [7]. The initial concentration of vitamin C (calculated as the sum of AA and DHAA) of all the treated drinks was not significantly different (7 mg per 100 mL on average). Nevertheless, when monitoring the concentration of vitamin C over 90 days of shelf life, independent from the treatment, a significant rapid decrease (*p* < 0.05) of this compound was found (Figure 1A,B). However, the degradation rate was noticeably higher for the HHP-treated samples than for the thermally treated beverages. Moreover, the higher losses occurred mainly during the first 7 days of storage, exhibiting losses of 45% and 85% on average at 4 and 20 °C, respectively, for both HHP treatments. On the other hand, 30% loss and 60% loss were found at 4 and 20 °C, respectively, for TP during the same period. Finally, the beverages presented a total degradation of vitamin C after 30 and 15 days for the HHP-treated drinks and after 45 and 60 days of storage for thermal treatment at 4 and 20 °C, respectively. Therefore, the storage temperature was identified as a critical factor affecting vitamin C breakdown, which is in agreement with previous authors [69,70]. In addition, another factor that may contribute to the loss of vitamin C is the mutual degradation between vitamin C and anthocyanins [71,72], as discussed below.

Regarding individual treatments, there were significant differences (*p* < 0.05) between the HHP-treated and thermally treated juices, since beverage processing by TP managed to extend a portion of the content of vitamin C up to 30 days more than juices subjected to HHP for both storage conditions during their shelf lives. Among other factors, these results may be due to the degradation of the active forms of vitamin C (AA and DHA) by enzymes with oxidase activity [73,74], such as peroxidase (POD), polyphenol oxidase (PPO), and particularly ascorbate oxidase (AO), which catalyze ascorbic acid oxidation, playing a major role in oxidizing ascorbic acid to dehydroascorbic acid (which is likewise rapidly oxidized to diketogulonic acid, the inactive form of vitamin C) in the early stages of storage in processed foods [75,76]. In this regard, HHP would not degrade some of these enzymes in citrus juices [77], unlike TP which, for temperatures over 80 °C, is capable of achieving a greater degree of inactivation of the oxidative enzymes [78,79]. Nevertheless, these results differ from other authors in the literature [80,81], who previously reported better conservation of vitamin C in HHP-treated juices than in thermally treated ones in fruit- and vegetable-based juices.

It is noteworthy that no differences were found between both HPP treatments.

### 3.5. Effect of HHP and TP Treatments on Phenolic Composition during Storage

#### 3.5.1. Flavanones

Regarding the flavanones of the citrus-maqui beverage, they were provided by citrus juices, being the most abundant eriocitrin (eriodyctiol 7-*O*-rutinoside), narirutin (naringenin 7-*O*-rutinoside), and hesperidin (hesperetin 7-*O*-rutinoside), characterized in preliminary studies by Salar et al. [7]. The total content of flavanones at the beginning of the beverage’s shelf life was 15.05 mg/100 mL on average for both HHP treatments and 23.33 mg/100 mL for TP.

**Figure 2:**
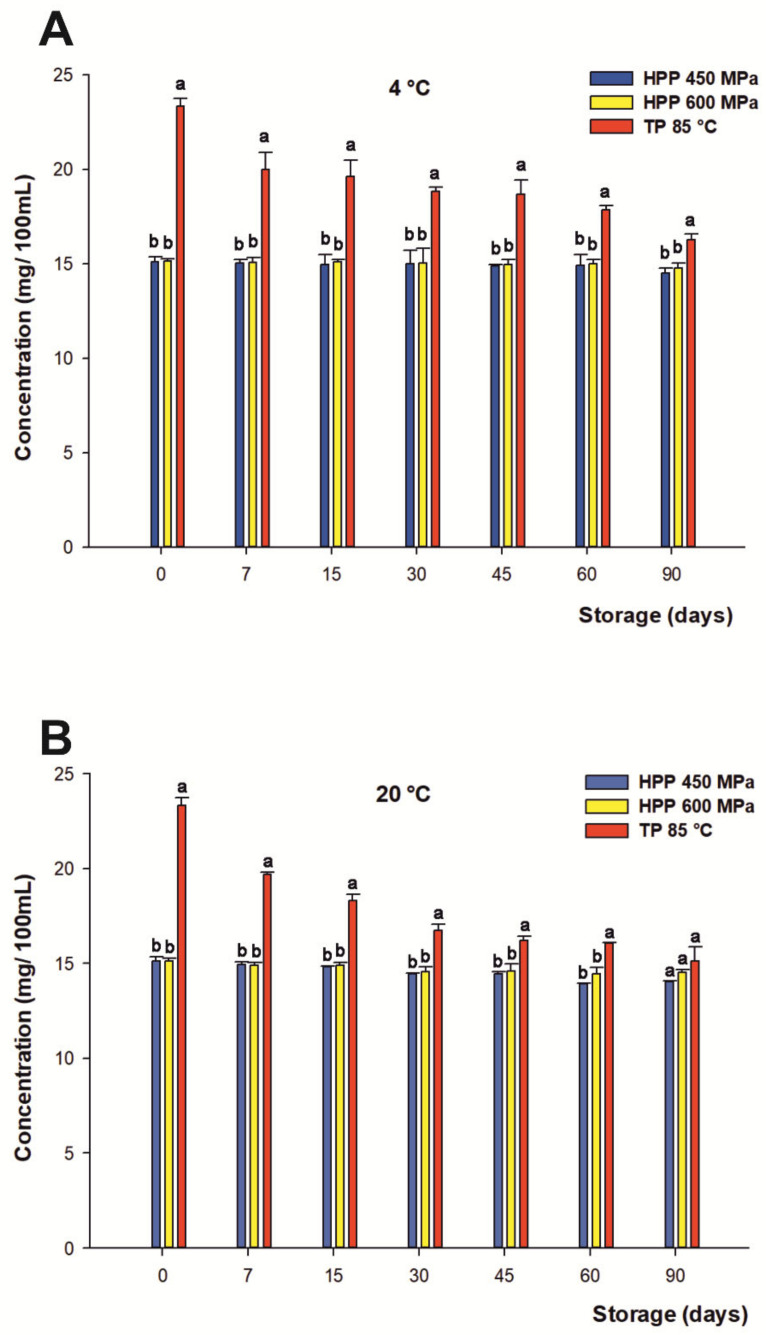
Changes of content in the total flavanones (mg/100 mL) of juices subjected to high hydrostatic pressure (HHP— 450 and HHP —600 MPa) and thermal pasteurization (TP— 85 °C), measured during storage for 90 days in refrigerated conditions at 4 °C (**A**) and 20 °C (**B**) under darkness conditions. Bars with different lowercase letters within each time point were different statistically at *p* < 0.05 according with the analysis of variance (ANOVA) and Tukey’s multiple range test.

Related to the variations in the content of total flavanones over the storage period, significant differences (*p* < 0.05) were found between both treatments submitted to HHP compared with TP (Figure 2A,B). In this frame, the HHP-processed juices managed to remain completely constant in their initial concentrations of flavanones over 90 days of storage at 4 °C, with a negligible final loss by 3% on average. Similar protective effects on the profile of flavanones in citrus juices processed by HHP in cold storage have been reported by Sanchez-Moreno et al. [82]. On the contrary, Plaza et al. pointed out important losses of 50% in the flavanone content just after 20 days of refrigerated storage in orange juice [83]. Aside from that, drinks processed by TP underwent losses of 14.3% just after 7 days, with a progressive degradation of the original content of flavanones during their shelf lives, reaching losses up to 30% at the end of refrigerated storage according previous descriptions in the literature [84].

On the other hand, the samples stored at room temperature followed a similar trend under HHP heat treatment with respect to cold storage, even if a final 6% loss was found for both HHP treatments on average, and for TP (35%) at the end of storage. This indicates that even if a higher concentration of these compounds is reached after treatment, phenolic compounds are more stable after both HHP treatments. Again, there were no significant differences between both HPP conditions.

Moreover, considering the contribution of individual flavanones to the total concentration of the phenolic compounds, neither *O*-tryglycosil-naringenin nor eriocitrin and narirutin displayed significant losses over 90 days of storage (data not shown), whereas hesperidin was quite affected under all processing treatments, as previously reported [8,85]. Overall, the results obtained in this study showed clearly that the total content of flavanones in the citrus-maqui juices was closely dependent on the processing treatment and subsequent storage conditions.

#### 3.5.2. Anthocyanins

The range of anthocyanins present in the citrus-maqui beverages was due to *A. Chilensis* [8], widely described and characterized in previous studies by Salar et al. [7]. In this connection, the initial content of the total anthocyanins of the juices recorded at the beginning of the storage period was 16.04 mg/100 mL on average for both HHP treatments and 18.37 mg/100 mL for TP. In the present study, the concentration of total anthocyanins remained constant during the first 15 days of storage at 4 °C for all processing treatments. These results are in contrast to some studies on berry juices, which reported higher losses for anthocyanins (over 30–60%) only 9 days after processing for samples submitted to HHP and storage at refrigerated temperatures [86,87].

**Figure 3:**
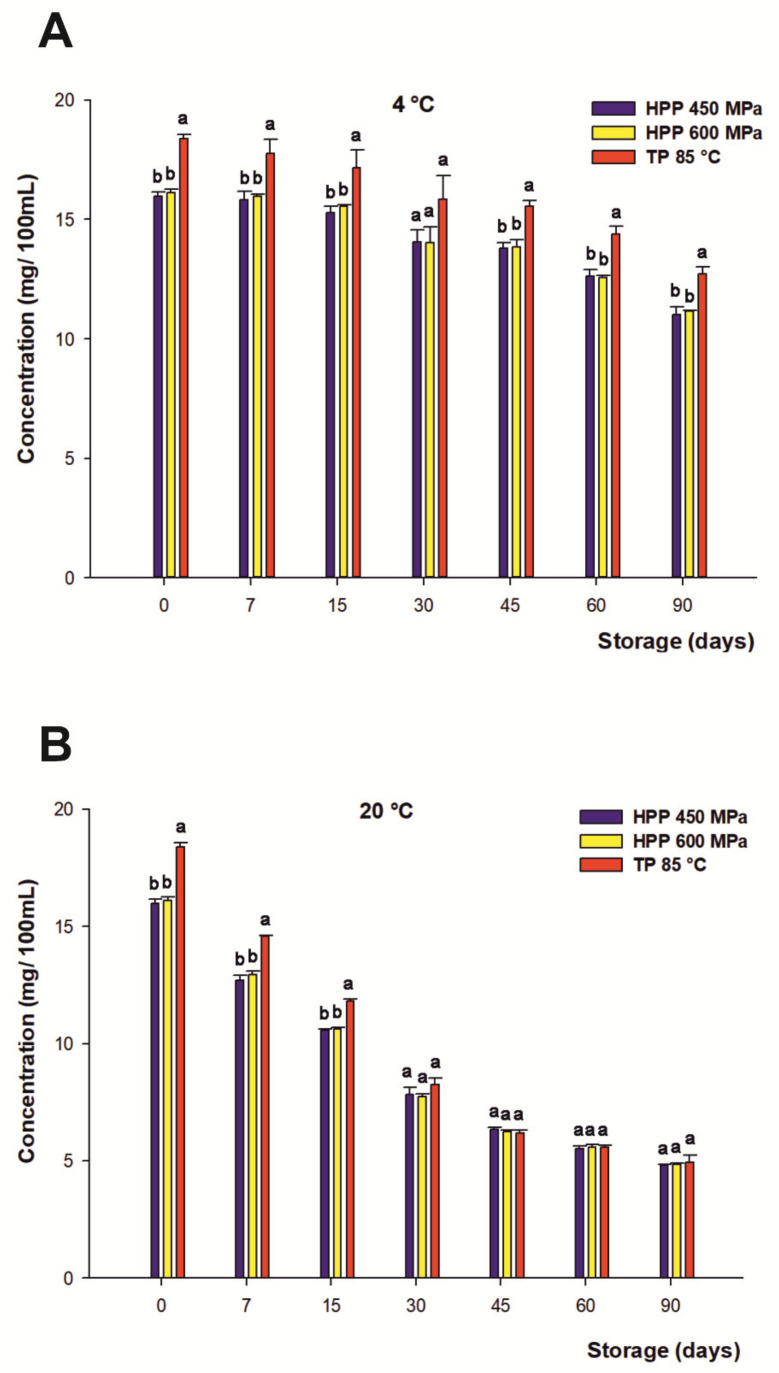
Changes in content of total anthocyanins (mg/100 mL) of juices subjected to high hydrostatic pressure (HHP—450 and HHP—600 MPa) and thermal pasteurization (TP—85 °C), measured during storage for 90 days in refrigerated conditions at 4 °C (**A**) and 20 °C (**B**) under darkness conditions. Bars with different lowercase letters within each time point were different statistically at *p* < 0.05 according to the analysis of variance (ANOVA) and Tukey’s multiple range test.

After the first fortnight of the storage period, the rate of anthocyanin degradation was gradual for all treatments, decreasing with time and without significant differences between both HHP processing treatments, reaching 30% loss on average at the end of the beverage’s shelf life regardless of the treatment under refrigerated storage at 4 °C (Figure 3A). These findings differ from other previous studies with juices containing anthocyanins, which have reported a higher final concentration of these phenolic compounds in HHP-treated drinks versus thermally treated ones during refrigerated storage [88]. On the other hand, the loss of anthocyanins in those samples stored at 20 °C (Figure 3B) displayed a higher degradation in anthocyanin content against those stored at 4 °C. This degradation was gradual, even if slower for TP during the first 15 days, reaching a 71% loss on average after 90 days for both the HHP and TP samples. Therefore, the degradation rate of the total anthocyanins was significantly accelerated with increasing storage temperatures. In this regard, some authors have described similar results in previous studies that reported a negative relationship between the storage temperature and the degradation of these colored flavonoids [89,90,91,92].

Moreover, anthocyanin stability in plant-based products depends on the interaction of various factors such as temperature, light, pH, presence of oxygen, metal ions, and solvents, among others [93,94]. In this sense, the mechanism involved in condensation reactions between ascorbic acid and anthocyanins [95] or the reaction of anthocyanins with free radicals generated by the degradation of ascorbic acid [71,72,96] could contribute to anthocyanin breakdown. It has also been reported by Castañeda-Ovando et al. that anthocyanin degradation takes place through the reactions of oxidation and condensation with other phenolic compounds, generating colorless compounds [97], even though the residual enzymatic activity of endogenous enzymes such as polyphenol oxidase (PPO), β-glucosidase(β-GLC), or peroxidase (POD) present in vegetable- and fruit-based beverages during storage could give rise to anthocyanins pigment degradation as well [98,99,100].

### 3.6. Color Changes of Juices during Storage

The color changes during 90 days of shelf life at 4 and 20 °C were determined by measuring the CIEL*a*b* color parameters. The reddish coloration of the new citrus-maqui blend was due to the total content of anthocyanins, which contribute to providing a more attractive appearance in the beverages for consumers.

Regarding the lightness (CIEL*) value, statistically significant differences were found between individual treatments (*p* < 0.05) throughout the storage period (Table 5 and Table 6). Overall, in the present study, the CIEL* value tended to increase independently of the preservation treatments and the storage temperatures. However, this increase was more evident for those beverages processed with HHP technology, being more pronounced for those processed at 600 MPa than at 450 MPa and stored at 20 °C. This is in accordance with previous studies but in contrast to other studies of juices rich in anthocyanins, which rose to create darker beverages during storage after processing by HHP [101,102,103] and which could most likely be attributed to distinct processing parameters and varied food arrays. On the other hand, the unequal rise of the CIEL* values among individual treatments could be due to the varying and usually low rate of enzymatic inactivation achieved by this emerging preservation technology in the processing of fruit- and vegetable-based beverages [44,104]. In this regard, the remaining residual enzyme activity of enzymes such as pectinases (pectin methyl esterase, polygalacturonase, and pectate lyase) in citrus-based juices can lead to destabilization of the cloud as a consequence of the sediment of cloud particles in the form of calcium pectate complexes [105,106], which may involve higher CIEL* values and a higher degree of clarification of citrus-based beverage processing by HHP during storage [67]. In this frame, some authors have described HHP processing at room temperature in vegetable- and fruit-based beverages as having limited effectiveness toward the inactivation of the main plant-based endogenous enzymes [77,107].

Furthermore, an overall trend to decrease redness (CIEa*) was observed for most of the samples as the shelf life increased (Table 5 and Table 6), which was associated with the degradation in total anthocyanins, in agreement with previous studies [94,108]. However, it is important to point out that this evolution in the CIEa* value was moved significantly toward the positive direction during storage in both HHP treatments when stored at 4 °C, being slightly higher for HHP-treated samples at 600 MPa, compared with thermal treatment. The increase in the CIEa* value observed in HHP-treated beverages stored at 4 °C could be associated with the breakdown of the citrus beverage cloud, owing to the formation and sedimentation of suspended particles (pectate complexes) in citrus-based juices [109], as previously mentioned. This breakdown may be due to the residual enzyme activity and supplementarily enhanced by a low storage temperature, which could contribute to faster particle precipitation [110], providing a lighter bright reddish color in the citrus-maqui drinks for those treated with HHP methods. It is also important to notice that the attractive reddish coloration of the beverages remained quite stable for 90 days for all treatments. Therefore, slight variations regarding the reddish color of the drinks over time could be associated to the formation of other newly colored polymers by co-pigmentation between anthocyanins and different phenolic compounds (flavonols, ferrulic acid, flavones, etc.) that could mask detrimental color losses or variations [111,112,113].

Moreover, slight changes were observed in the yellowness (CIEb* value), as this parameter increased along with the shelf life time for most of the drinks, mainly in HHP-treated beverages at 450 MPa relative to the HHP-treated ones at 600 MPa or under thermal treatment (Table 5 and Table 6). With respect to the Chroma and Hue angle parameters, an overall increase was noted for all treatments and temperatures, indicating a numerical browning inclination, which could barely be detected by the naked eye.

On the other hand, the total color difference (ΔE) increased along the 90 days of storage (Table 5 and Table 6), indicating significant color changes between individual treatments in the citrus-maqui juices. In the current study, both HHP treatments led to a higher increase in ΔE, significantly more emphasized in those beverages treated at 600 MPa compared with those under thermal treatment and being more accentuated in those samples stored at 20 °C. With regard to the foregoing, the drinks maintained a stable color up to 15 days of storage without visual alterations, and visual differences were only appreciated by the human eye when the ΔE values surpassed 12 units. These results are in contrast with those found by other authors in red fruit juices [114,115], which exposed that noticeable visual differences for ΔE could be appreciated by the naked eye for value differences of just three units. In the present study, the differences among treatments in the ΔE values were mainly due to outstandingly higher CIEL* values in those HHP-treated samples over time compared with the TP-treated samples, probably due to the cloud loss, as previously discussed.

**Figure 4:**
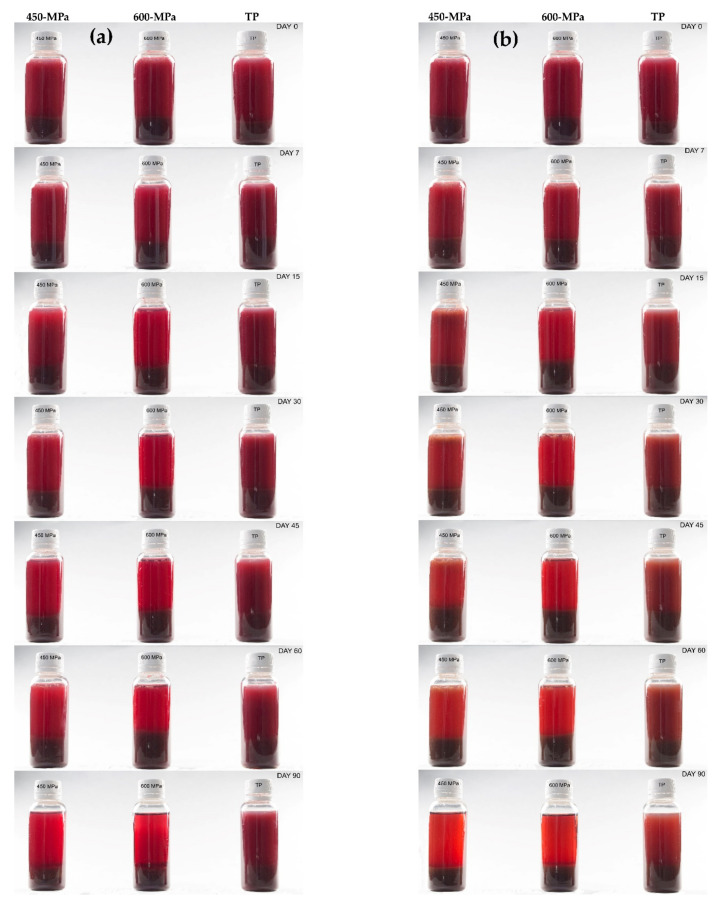
Visual appearance of citrus-maqui based beverages processed by HHP at different pressure levels (HHP—450 MPa and HHP—600MPa) and TP—85°C over 90 days of storage at 4°C (**a**) and 20°C (**b**).

On the whole, the shelf life had a significant impact on the color parameters monitored for beverages processed under either HHP or TP, although those samples processed by TP retained their original colors better than HHP, in accordance with previous reports [65,116]. Finally, it should be noticed that despite most consumers preferring fresh, cloudy juices without signs of sedimentation [117], in the present study, cloud loss in the citrus-maqui beverages provided a more intense and bright reddish color over storage time, as shown in Figure 4a,b, which could result in a more visually attractive commercial beverage for potential consumers [118].

## 4. Conclusions

In this study, the application of HHP showed interesting results related to the microbial safety and stability of all parameters analyzed in the citrus-maqui beverages. Neither the processing nor storage conditions had an impact on the physicochemical parameters. Moreover, both HHP and TP guaranteed the microbiological safety throughout the shelf life. Regarding bioactive compounds, although TP promoted an increase in the content of flavonoids immediately after processing, HHP displayed a higher protective effect on bioactive flavonoids (flavanones and anthocyanins), giving rise to a lower rate of degradation during storage and preserving the healthy properties for the beverages. On the other hand, vitamin C underwent a rapid degradation for all processing conditions. Aside from that, the color parameters remained rather stable for all treated samples, keeping an attractive reddish coloration, although the use of HHP and refrigeration better preserved the intensity of the color during storage. It is noteworthy that color was the only studied parameter slightly affected by the HPP conditions (450 or 600 MPa). Finally, the storage temperature was shown to be the most critical parameter for the degradation of all the studied bioactive compounds, with 4 °C being the most suitable temperature for storage. Due to these results, HHP could be considered an effective alternative to conventional TP in the food industry for the production of high functional quality fruit-based beverages.

## Figures and Tables

**Table 1:** Codification of samples included in the experimental design.

Code	Beverage and Storage Conditions
Control	Untreated sample
HHP—450 MPa 4	Beverage subjected to high hydrostatic pressure (450 MPa) stored at 4 °C under darkness conditions
HHP— 450 MPa 20	Beverage subjected to high hydrostatic pressure (450 MPa) stored at 20 °C under darkness conditions
HHP— 600 MPa 4	Beverage subjected to high hydrostatic pressure (600 MPa) stored at 4 °C under darkness conditions
HHP— 600 MPa 20	Beverage subjected to high hydrostatic pressure (600 MPa) stored at 20 °C under darkness conditions
TP—85 °C 4	Beverage subjected to thermal pasteurization stored at 4 °C under darkness conditions
TP—85 °C 20	Beverage subjected to thermal pasteurization stored at 20 °C under darkness conditions

**Table 2:** Physicochemical parameters and antioxidant biomolecules of non-processed, pressurized, and pasteurized citrus-maqui beverages at zero time of storage.

Condition ^Z^	Physicochemical Parameters				Bioactive Compounds (mg/100 mL)		
	Color (ΔE)	TSS (°Brix)	pH	TA (g CA/100 mL)	Anthocyanins	Flavanones	Vitamin C
Non-processed	0.00c ^Y^	13.60 a	3.41 b	2.95 a	16.54 b	14.99 b	10.90 a
HHP—450 MPa	1.39bc	13.60 a	3.45 a	2.91 a	15.98 c	15.11 b	6.03 b
HHP—600 MPa	1.82b	13.60 a	3.40 b	2.95 a	16.1 c	15.14 b	7.49 b
TP—85 °C	3.59a	13.60 a	3.41 b	2.95 a	18.37 a	23.33 a	7.79 b
LSD (*p* < 0.05)	1.02	<0.01	0.03	<0.01	0.31	0.48	1.70
*p*-value	*** ^X^	N.s.	*	N.s.	***	***	**

^Z^ HHP— 450 MPa, processing at 450 MPa/3 min; HHP— 600 MPa, processing at 600 MPa/3 min; TP— 85 °C, thermal pasteurization at 85 °C/15 s. ^Y^ Data (means) within each column, with values followed by different letters for each processing condition, are significantly different at *p* < 0.05 according to the analysis of variance (ANOVA) and Tukey’s multiple range test. ^X^ Significant at * (*p*< 0.05), ** (*p* < 0.01), and *** (*p* < 0.001) according to a paired *t*-test. N.s. = not significant differences.

**Table 3:** Microbiological quality of non-processed, pressurized, and pasteurized citrus-maqui beverages at zero time of storage.

Condition ^Z^	Microbiological Count (CFU/mL)				
	Aerobic Mesophilic Bacteria	Aerobic Psycrophilic Bacteria	Molds and Yeast	Enterobacteriae	Lactic Acid Bacteria
Non-processed	80 ± 5	<10 ^Y^	<100 ^Y^	<10 ^Y^	<10 ^Y^
HHP—450 MPa	<10 ^Y^	<10 ^Y^	<100 ^Y^	<10 ^Y^	<10 ^Y^
HHP—600 MPa	<10 ^Y^	<10 ^Y^	<100 ^Y^	<10 ^Y^	<10 ^Y^
TP—85°C	<10 ^Y^	<10 ^Y^	<100 ^Y^	<10 ^Y^	<10 ^Y^

^Z^ HHP— 450 MPa, high hydrostatic pressure at 450 MPa/3 min; HHP— 600 MPa, high hydrostatic pressure at 600 MPa/3 min; TP— 85°C, thermal pasteurization at 85 °C/15 s. ^Y^ Values below the detection limit for aerobic mesophilic and psychrophilic bacteria, Enterobacteriaceae and lactic acid bacteria (<10 CFU/mL), and molds and yeasts (<100 CFU/mL).

**Table 4:** pH, titratable acidity (TA), and total soluble solids (TSS) measured at day 0 (initial) and after 90 days of storage (final) for beverages subjected to HHP and TP and stored under two different conditions.

Condition ^Z^	TSS (°Brix)			pH			TA (g CA/100 mL)		
	Initial	Final	*p*-Value	Initial	Final	*p*-Value	Initial	Final	*p*-Value
HHP—450 MPa 4	13.60 a ^Y^	14.20 a	*** ^X^	3.45 a	3.41 a	*	2.91 a	3.00 a	*
HHP—600 MPa 4	13.60 a	14.20 a	***	3.40 b	3.41 a	N.s.	2.95 a	3.00 a	N.s.
TP—85°C 4	13.60 a	14.20 a	***	3.41 b	3.41 a	N.s.	2.95 a	2.98 a	N.s.
LSD (*p* <0.05)	<0.01	<0.01		0.03	<0.01		<0.01	<0.01	
*p*-value	N.s.	N.s.		*	N.s.		N.s.	N.s.	
HHP—450 MPa 20	13.60 a	14.20 a	***	3.45 a	3.40 a	*	2.91 a	2.98 a	*
HHP —450 MPa 20	13.60 a	14.20 a	***	3.40 b	3.40 a	N.s.	2.95 a	3.00 a	*
TP— 85°C 20	13.60 a	14.20 a	***	3.41 b	3.41 a	N.s.	2.95 a	2.99 a	*
LSD (*p* <0.05)	<0.01	<0.01		0.03	<0.01		<0.01	<0.01	
*p*-value	N.s.	N.s.		*	N.s.		N.s.	N.s.	

^Z^ HHP—450MPa 4, high hydrostatic pressure at 450 MPa/4 °C; HHP—600MPa 4, high hydrostatic pressure at 600 Pa/4°C; TP—85°C 4, thermal pasteurization at 85 °C/4 °C; HHP—450 MPa 20, high hydrostatic pressure at 450 MPa/20°C; HHP—600 MPa 20, high hydrostatic pressure at 600 MPa/20 °C; TP—85°C 20, thermal pasteurization at 85 °C/20 °C,. Initial and final values were significantly different according to a paired *t*-test at * (*p* < 0.05), ** (*p* < 0.01), and *** (*p* < 0.001). ^Y^ For the data (means) within each column, values followed by different letters for each processing condition are significantly different at *p* < 0.05 according to the analysis of variance (ANOVA) and Tukey’s multiple range test. ^X^ Significant at * (*p* < 0.05) and *** (*p* < 0.001) according to a paired *t*-test. N.s. = not significant differences.

**Table 5:** Stability of CIEL*a*b* values in beverages stored at 4 °C.

Parameter	Storage (Days)	HHP-450 MPa	HHP-600 MPa	TP-85 °C	LSD (*p* < 0.001)
^w^ CIEL*	0	23.39 aB ^Z^	22.55 aAB	21.38 deA	1.04
	7	23.53 aB	24.74 bC	19.57 abA	0.80
	15	24.64 bB	33.54 cC	19.72 aA	0.51
	30	30.96 cB	38.13 dC	20.54 bcA	0.49
	45	33.58 dB	39.08 eC	20.31 abA	0.13
	60	33.49 dB	40.31 fC	21.23 cA	0.41
	90	34.11 dB	41.12 gC	21.97 eA	0.63
	LSD (*p* < 0.001)	0.69	0.41	0.46	
CIEa*	0	47.87 aB	47.48 aAB	46.78 bA	0.86
	7	47.86 aB	48.93 bC	44.80 aA	0.79
	15	48.40 aB	56.53 cC	45.06 aA	0.50
	30	53.48 cB	59.01 eC	45.26 aA	0.49
	45	54.83 dB	58.50 eC	44.60 aA	0.40
	60	53.35 cB	57.49 dC	45.03 aA	0.21
	90	52.39 bB	56.42 cC	44.89 aA	0.59
	LSD (*p* < 0.001)	0.57	0.41	0.43	
CIEb*	0	37.15 aB	36.30 cAB	34.82 cdA	1.41
	7	37.39 aB	38.54 eC	32.41 aA	0.80
	15	38.52 bB	37.85 dB	32.63 aA	0.59
	30	40.95 deC	32.68 aA	33.85 abcB	0.28
	45	39.73 cC	32.73 aA	33.59 abB	0.53
	60	40.29 cdC	32.81 aA	34.71 bcdB	0.60
	90	41.70 eC	33.95 bA	35.79 dB	0.49
	LSD (*p* < 0.001)	0.68	0.41	0.76	
Chroma	0	60.60 aB	59.76 aAB	58.31 cA	1.52
	7	60.73 abB	62.28 bC	55.30 aA	1.10
	15	61.86 bB	68.03 dfC	55.63 aA	0.73
	30	67.36 cB	67.46 efB	56.52 abA	0.25
	45	67.71 cC	67.04 deB	55.84 aA	0.14
	60	66.85 cC	66.20 cdB	56.86 abA	0.52
	90	66.96 cA	65.85 cB	57.41 bcA	0.32
	LSD (*p* < 0.001)	0.77	0.54	0.78	
Hue angle	0	37.81 bcB	37.40 fAB	36.66 bcA	0.62
	7	38.00 cdB	38.22 eB	35.88 abA	0.19
	15	38.51 bdC	33.80 dA	35.91 aB	0.27
	30	37.44 bcC	28.98 aA	36.79 bcB	0.02
	45	35.92 aB	29.23 aA	36.95 cC	0.14
	60	37.06 bB	29.71 bA	37.62 dB	0.66
	90	38.52 dB	31.04 cA	38.56 eB	0.59
	LSD (*p* < 0.001)	0.51	0.18	0.39	
ΔE	0	0.00 a	0.00 a	0.00 a	<0.01
	7	0.47 aA	3.45 bB	2.86 cdB	1.26
	15	1.93 bA	14.32 cC	3.24 dB	0.90
	30	10.16 cB	19.72 dC	1.99 bcA	0.28
	45	12.61 dB	20.18 dC	2.72 cdA	0.13
	60	12.34 dB	20.68 dC	1.76 bA	0.31
	90	12.49 dB	20.74 dC	2.22 bcA	0.64
	LSD (*p* < 0.001)	0.48	0.66	0.57	

^Z^ Means (*n* = 3) within a column followed by a different letter (storage time point comparison, lowercase letter) or within a row (treatment comparison, capital letter) are significantly different at *p* < 0.001. ^W^ CIEL* = lightness; CIEa* = redness; CIEb* = yellowness; ΔE = difference or distance between two colors.

**Table 6:** Stability of CIEL*a*b* values in beverages stored at 20 °C.

Parameter	Storage (Days)	HHP-450 MPa	HHP-600 MPa	TP-85 °C	LSD (*p* < 0.001)
CIEL*	0	23.39 aB ^Z^	22.55 aAB	21.38 bA	1.04
	7	24.84 bB	31.80 bC	20.51 aA	0.74
	15	28.84 cB	37.44 cC	22.23 bA	0.51
	30	32.55 dB	40.13 dC	22.31 bA	0.64
	45	37.06 eB	43.61 eC	23.86 cA	0.43
	60	40.07 gB	45.71 fC	23.66 cA	1.59
	90	38.52 fB	47.81 gC	24.13 cA	0.11
	LSD (*p <* 0.001)	0.69	0.92	0.66	
CIEa*	0	47.87 dB	47.48 dAB	46.78 eA	0.86
	7	47.08 cdB	51.89 fC	44.36 dA	0.68
	15	47.75 dB	53.61 gC	44.15 dA	0.38
	30	46.34 cB	49.76 eC	41.51 cA	0.24
	45	45.19 bB	46.88 cC	40.19 bA	0.30
	60	44.60 bB	45.12 bC	39.41 aA	0.18
	90	42.19 aB	43.92 aC	38.92 aA	0.32
	LSD (*p <* 0.001)	0.51	0.19	0.49	
CIEb*	0	37.17 aB	36.30 aAB	34.82 abA	1.41
	7	39.14 bB	41.84 cC	33.76 aA	0.86
	15	43.15 cB	40.34 bC	36.19 bcA	0.64
	30	46.56 dC	44.07 dB	36.57 cA	0.73
	45	48.31 eC	44.76 dB	38.78 dA	0.55
	60	48.68 eC	45.63 eB	38.64 dA	0.91
	90	50.36 fC	46.23 eB	39.24 dA	0.14
	LSD (*p <* 0.001)	0.67	0.54	0.92	
Chroma	0	60.60 aB	59.76 aAB	58.31 cA	1.52
	7	61.22 aB	66.66 deC	55.75 abA	1.05
	15	64.35 bB	67.10 eC	57.09 bcA	0.69
	30	65.69 cB	66.48 dB	55.32 aA	0.63
	45	66.15 cA	64.81 cB	55.85 abA	0.58
	60	66.03 cC	64.17 bcB	55.19 aA	0.60
	90	65.70 cC	63.77 bB	55.26 aA	0.32
	LSD (*p <* 0.001)	0.81	0.39	0.93	
Hue angle	0	37.81 aB	37.40 aAB	36.66 aA	0.62
	7	39.74 bC	38.88 bB	37.27 aA	0.28
	15	42.10 cC	36.96 aA	39.45 bB	0.66
	30	45.14 dB	41.53 cA	41.38 cA	0.46
	45	46.92 eB	43.67 dA	43.98 dA	0.25
	60	47.50 fC	45.32 eB	44.43 dA	0.63
	90	50.04 gC	46.46 fB	45.24 eA	0.17
	LSD (*p <* 0.001)	0.21	0.37	0.47	
ΔE	0	0.00 a	0.00 a	0.00 a	<0.01
	7	2.63 bA	11.64 bB	2.80 bA	0.97
	15	8.10 cB	16.60 cC	3.14 bA	0.33
	30	13.23 dB	19.35 dC	5.67 cA	0.40
	45	17.85 eB	22.70 eC	8.09 dA	0.19
	60	20.53 fB	25.10 fC	8.61 dA	1.10
	90	20.88 fB	27.37 gC	9.46 eA	0.19
	LSD (*p <* 0.001)	0.47	0.64	0.43	

^Z^ Means (*n* = 3) within a column followed by a different letter (storage time point comparison, lowercase letter) or within a row (treatment comparison, capital letter) are significantly different at *p <* 0.001.

## Data Availability

Not applicable.
